# In vitro induction of odontogenic activity of human dental pulp stem cells by white Portland cement enriched with zirconium oxide and zinc oxide components

**DOI:** 10.15171/joddd.2019.001

**Published:** 2019-04-24

**Authors:** Saeed Rahimi, Sadegh Salarinasab, Negin Ghasemi, Reza Rahbarghazi, Shahriar Shahi, Amin Salem milani, Baharak Divband, Paria Davoudi

**Affiliations:** ^1^Dental and Periodontal Research Center, Department of Endodontics, Faculty of Dentistry, Tabriz university of Medical Sciences, Tabriz, Iran; ^2^Department of Biochemistry and Clinical Laboratories, Faculty of Medicine, Tabriz University of Medical Sciences, Tabriz, Iran; ^3^Department of Applied Cell Sciences, Faculty of Advanced Medical Sciences, Tabriz University of Medical Sciences, Tabriz, Iran; ^4^Stem Cell Research Center, Tabriz University of Medical Sciences, Tabriz, Iran; ^5^Department of Inorganic Chemistry, Faculty of Chemistry, University of Tabriz, Tabriz, Iran; ^6^Department of Endodontics, Faculty of Dentistry, Tabriz University of Medical Sciences, Tabriz, Iran

**Keywords:** Human dental pulp stem cells, odontogenic differentiation, white Portland cement, zinc oxide nanoparticles, zirconium oxide nanoparticles

## Abstract

***Background***. The aim of this in vitro study was to investigate the effect of zinc oxide (ZnO) and zirconium oxide (ZrO_2_) microparticles (MPs) and nanoparticles (NPs) in combination with white Portland cement (WPC) on odontogenic capacity of human dental pulp stem cells over a period of 21 days.

***Methods***. Synthesized ZnO and ZrO_2_ particles were characterized using scanning electron microscopy and transmission electron microscopy. The viability of human dental pulp stem cells was measured by a 3-(4,5-dimethylthiazolyl-2-yl)-2,5- diphenyltetrazolium bromide assay at 7-, 14- and 21-day intervals after seeding on WPC disks enriched with ZnO and ZrO_2_ MPs and NPs. Odontogenic potential of ZnO and ZrO_2_ particles in combination with WPC was investigated by alkaline phosphatase (ALP) activity and ionized calcium level of supernatant culture media at different time intervals. Data were analyzed using one-way ANOVA and post hoc Tukey tests.

***Results***. All the materials exhibited cell viability over a 21-day period, except for WPC with ZnO NPs on day 7, although it was not statistically significant (P>0.05). The ALP activity and ionized calcium level increased in all the groups compared to the control group (P<0.05). ZnO NPs had superior effect on odontogenic activity and calcium ion release compared to ZnO MPs (P=0.046). There was no significant difference between ZrO_2_ MPs and NPs in odontogenic activity (P>0.05).

***Conclusion***. WPC enriched with ZnO and ZrO_2_ increased ALP activity and calcium ion release of human dental pulp stem cells over a period of 21 days in vitro.

## Introduction

Manipulation and regulation of stem cell (SC) dynamics, especially dental SCs, is an inevitable factor in regenerative procedures.^1^ Human dental pulp SCs (HDPSCs), residing in the dental pulp, can easily trans-differentiate into different cell types notably odontoblasts and osteoblasts.^2^ HDPSCs have a potential to differentiate into mature cell type by expressing factors such as odontoblast differentiation markers, dentin sialophosphoprotein (DSPP) and dentin matrix protein 1 (DMP-1). Commensurate with this statement, alkaline phosphatase (ALP) activity is also induced during odontogenic differentiation of HDPSCs, with a crucial role in mineralization and acquisition of dentin-like structures.^3^

Considering the distinct structure of dentin and the existence of hard tissues, it is essential to deliver SCs to the injured site by using appropriate scaffolds protecting transplanted cells from mechanical insult while providing a 3D structure for normal cell arrangement and cell-to-cell communication.^4^ The applied scaffolds must be biocompatible with the ability to stimulate repair by formation of mineralized tissues. Provision of antimicrobial activity, sealing ability, dimensional stability and radiopacity are other properties required for the regeneration of the target sites.^5^

Currently, one of the most popular dental materials used in the field of regenerative endodontics is mineral trioxide aggregate (MTA), a derivate of Portland cement (PC), which is used extensively for the induction of bio-mineralization.^6^ Some disadvantages of MTA are prolonged therapeutic approach, difficult handling, dental discoloration and some cytotoxic effects due to the presence of bismuth oxide (BO) in its chemical structure as a radiopacifier.^7-12^ The pure PC alone could induce the expression of dentin-associated markers but it lacks radiopacity, making it inappropriate for clinical use.^13^ To circumvent these limitations, many suitable radiopacifying agents have been proposed to date, notably zirconium oxide (ZrO_2_) and zinc oxide (ZnO).^14,15^ Evidence has shown some biological activities for ZrO_2_ and ZnO particles such as calcium release, antibacterial effects and pH stability.^16-19^ Provision of nano- and/or micro-sized particles helps ZnO promote the functional action of osteoblasts while increasing the bactericidal effects.^20^ The construction of ZrO_2_ nanoparticles (NPs) was found to improve the physicochemical properties of PC.^17,21^

Thus, in this in vitro study, we aimed to investigate the effects of incorporation of ZnO and ZrO_2_ microparticles (MPs)/NPs into white PC (WPC) on the odontogenic capacity of HDPSCs over a period of 21 days.

## Methods

### Synthesis of Zirconium Oxide and Zinc Oxide Micro- and Nanoparticles

The synthesis of MPs and NPs was performed in the Laboratory of Nanotechnology, Chemistry Institute of Tabriz, Iran. To synthesize ZrO_2_ MPs, anhydrous ZrCl_4_ was heated at 700°C for 7 hours. ZrO_2_ NPs were also prepared by dissolving anhydrous ZrCl_4_ in 3M HCl solution at 40°C for 3 hours. Thereafter, 20 mL of ethylene glycol, as an esterification agent, was overlaid under vigorously stirring. The solution was stirred for a period of 12 hours, followed by heating to 80°C for 4 hours to form a yellow participate. Finally, the suspension was centrifuged at 10000 rpm, washed and calcined at 450°C for 5 hours.

In order to prepare ZnO MPs, zinc acetylacetonate hydrate was heated at 700°C for 7 hours. To synthesize ZnO NPs, zinc acetylacetonate hydrate was dissolved in 1M HNO_3_ solution and citric acid for 3 hours. Similar to the preparation of ZrO_2_ NPs, 20 mL of ethylene glycol was added to the solution, followed by the above-mentioned procedures.

### SEM and TEM Analysis of Synthesized Particles

**Figure 1:**
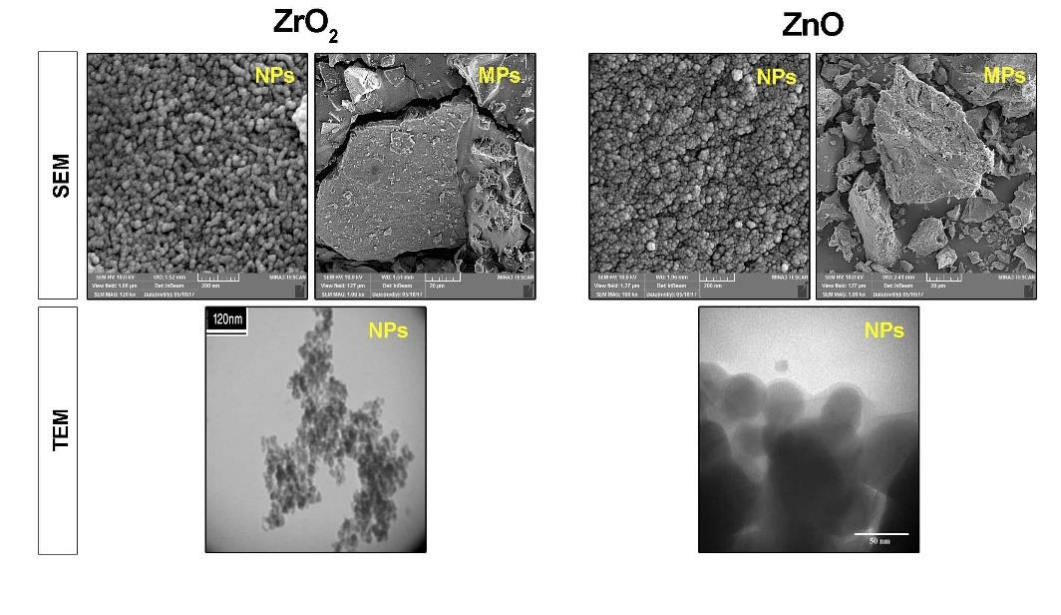
Representative images of ZrO2 and ZnO particles analyzed by SEM and TEM techniques.

The morphological features of the synthesized MPs and NPs were characterized using scanning electron microscopy (SEM; Model: MRIA3-FEG-SEM Tescan, Brno, Czech,) and transmission electron microscopy (Zeiss LEO 912 Omega, TEM) for NPs, respectively (Figure 1).

### Sample Preparation

In the current study, 6 experimental groups were established (Table 1). A ratio of 30% radiopacifier (the synthesized ZnO/ZrO_2_ components) and 70% WPC (Tehran Cement Co., Tehran, Iran) was used. The WPC+ZnO MPs, WPC+ZnO NPs, WPC+ZrO_2_ MPs and WPC+ ZrO_2_ NPs were manipulated using a powder-to-liquid (distilled water) ratio of 1 g per 0.33 mL, providing proper handling and consistency. Pure WPC was also manipulated with this ratio. All these manipulations were carried out under aseptic conditions.

**Table 1:** The experimental groups

**Group**	**Material**
**Control (Plastic surface)**	-
**WPC**	WPC
**WPC + ZnO MPs**	WPC (70%) + ZnO MPs (30%)
**WPC + ZnO NPs**	WPC (70%) + ZnO NPs (30%)
**WPC + ZrO**_2_ ** MPs**	WPC (70%) + ZrO_2_ MPs (30%)
**WPC + ZrO**_2_ **NPs**	WPC (70%) + ZrO_2_ NPs (30%)

White Portland cement (WPC); Zinc oxide (ZnO); Zirconium oxide (ZrO_2_); Microparticles (MPs); Nanoparticles (NPs)

Based on different analyses carried out in this experiment, disks with different diameters and 2 mm in thickness were prepared. To preserve the consistency, the disks were wrapped in pieces of moist gauze and incubated for 24 hours in a closed container. To perform cell culture and in vitro tests, disks were first sterilized using ethylene dioxide gas sterilization and then placed on the bottom surface of each well and washed two twice with phosphate-buffered saline before cell culture.

### Cell Culture

In this study, we used human dental pulp stem cell line (HDPSCs; Royan Institute, Tehran, Iran). HDPSCs were cultured and expanded in low-content glucose Dulbecco’s modified Eagle medium (DMEM/LG; Gibco), with 10% fetal bovine serum (FBS; Gibco) and 1% penicillin-streptomycin (Biosera). The culture plates were maintained at 37ºC in a humidified atmosphere with 5% CO_2_. The cells between passages 3 to 6 were subjected to various experiments.

### MTT Assay

The cytotoxic effect of materials was evaluated by using (4,5-dimethylthiazol-2-yl)-2,5-diphenyltetrazolium bromide colorimetric assay [Mosmann’s tetrazolium toxicity (MTT) assay] (Sigma Chemical Co., St Louis, MO, USA). The disks were placed on the bottom surface of each well of 96-well plates and 1 mL of DMEM/LG medium with 1% FBS containing 1×10^5^ HDPSCs was overlaid. After 7, 14 and 21 days, the supernatant was discarded and disks were incubated with 100 µL of MTT (5 mg/mL) for 4 hours. Thereafter, 50 µL of dimethyl sulfoxide (DMSO) were added and the absorbance was read by a microplate reader (BioTek) at a wavelength of 450 nm. The percentage of viable cells was represented as percentage of control.

### Alkaline Phosphatase Activity

In order to confirm the odontogenic/osteogenic capacity of treated cement on HDPSCs, ALP activity was measured at 7-, 14- and 21-day intervals after seeding on the materials. To this end, 1×10^5^ HDPSCs was added to each well of 24-well plates and the supernatant was collected from each group and centrifuged at 400 g for 5 minutes. Thereafter, ALP activity was calculated for each group by using an ELISA kit (Roche Hitachi 912, Germany). Finally, the amount of ALP activity was reported as IU/L.

### Calcium Ion Level

The supernatant of each well of 24-well plates after adding 1×10^5^ HDPSCs was collected and after centrifugation at 400 g for 5 minutes, the total calcium ion content was measured by using an AutoAnalizer (Roche Hitachi 912, Germany) on days 7, 14 and 21.

### Statistical Analysis

All in vitro analyses were performed in triplicates. Data were presented as mean ± SD and analyzed using one-way ANOVA with post hoc Tukey tests using SPSS 20.0 (SPSS Inc., Chicago, IL). The significant level was set at P<0.05.

## Results

### Cell Viability

**Figure 2:**
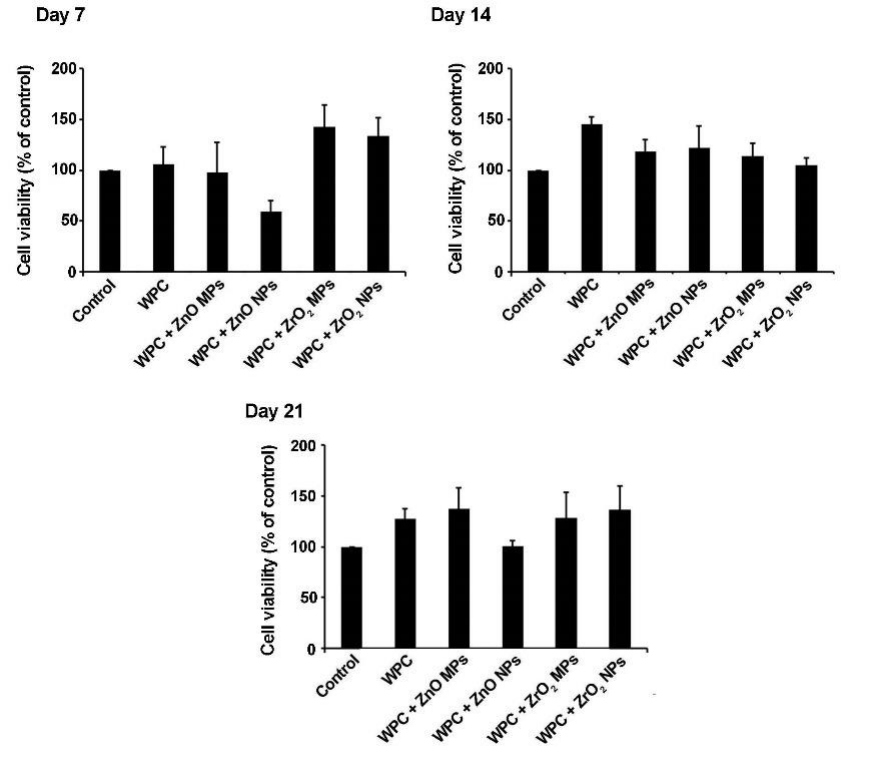
Detection of HDPSCs survival rates, plated on WPC enriched by ZrO2 and ZnO particles over a period of 21 days. In all the groups, except for WPC + ZnO NPs, an increased cell survival rate was evident. On day 21, the cell viability index in cells from the WPC + ZnO NP group was similar to the control group. These data showed that WPC substrate enriched by ZrO2 and ZnO particles was suitable for the expansion and culture of HDPSCs

According to data from MTT assay, increased cell viability was observed in pure WPC and WPC with ZnO MPs, ZrO_2_ MPs and NPs compared with the control group over a period of 21 days. Notably, WPC+ZnO NP group exhibited decreased cell survival rate on day 7 but it was not statistically significant (P>0.05), and this value reached levels higher than the matched-control on days 14 and 21 (Figure 2).

### Alkaline Phosphatase Activity

**Figure 3:**
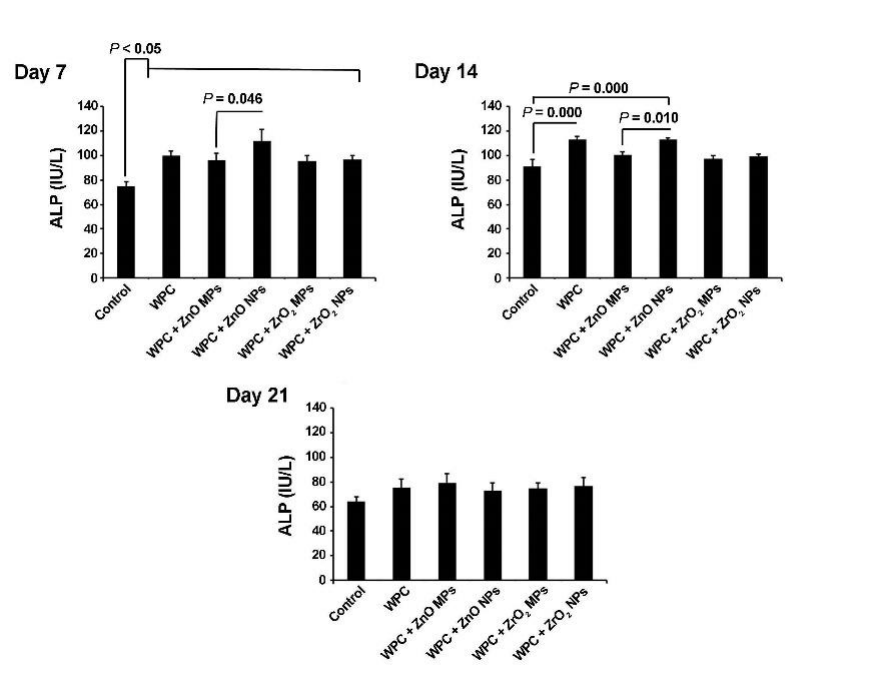
Monitoring odontoblastic property of WPC enriched by ZrO2 and ZnO particles on HDPSCs via the ALP activity. The ALP activity was found to increase at all the time intervals from different groups. This effect was more evident at 7- and 14-day intervals after cell culture compared to cells from day 21 (n=3) (one-way ANOVA with Tukey tests).

In light of ALP activity, the ELISA assay showed significant differences in ALP activity in groups over a period of 21 days (P<0.05). On day 7, there was a statistically significant ALP activity increase in all the groups in comparison with the control group. Compared to cells grown on WPC+ZnO MP surfaces, HDPSCs juxtaposition to WPC+ZnO NPs resulted in a superior ALP activity (P_day 7_ =0.046, P_day 14_ =0.01). No significant difference was observed in ALP activity between WPC+ZrO_2_ MP and WPC+ZrO_2_ NP groups (P>0.05). On day 14, significant differences in ALP activity were detected in WPC and WPC+ZnO NPs (P<0.05). The level of ALP activity decreased on day 21 in comparison with matched-groups of day 7. Although the level of ALP activity was more than the control group in all the groups 21 days after cell culture, it was not statistically significant (P>0.05; Figure 3).

### Ionized Calcium Level

**Figure 4:**
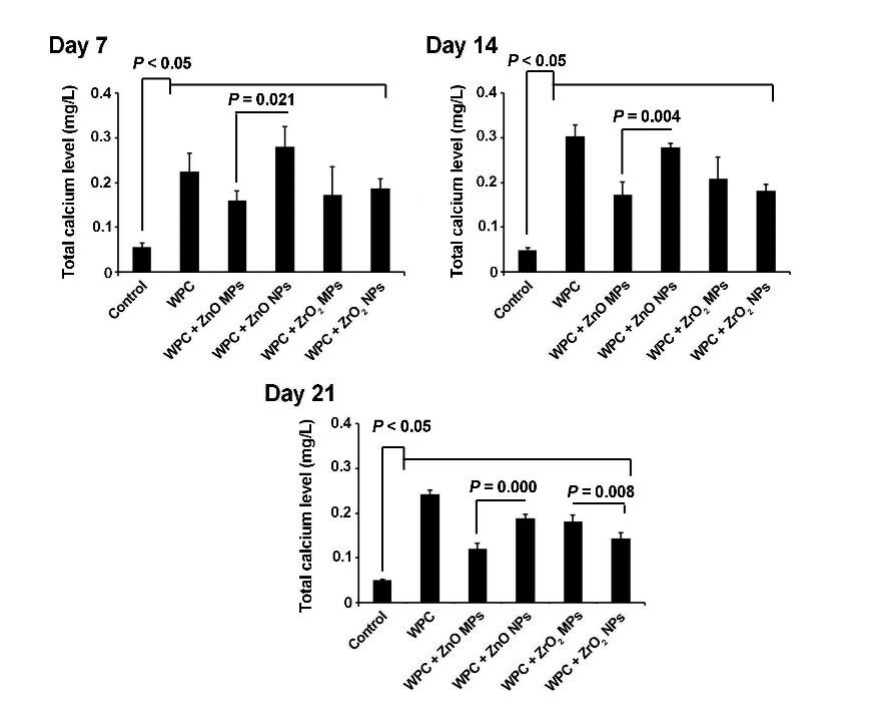
Ionized calcium level in the experiment groups. Total calcium level was shown to increase after cell plating on different substrates at all the time intervals (n=3) (one-way ANOVA with Tukey tests).

All the materials used in the current experiment exhibited an increased ionized calcium level in the supernatant in comparison with the control group at the 3 time intervals (P<0.05). On day 14, a superior effect of WPC+ZnO NPs and WPC alone was observed on the ionized calcium content (P<0.05). Based on our data, the calcium level decreased but remained significant compared with cells from 7- and 14-day time intervals (Figure 4).

## Discussion

Stimulation of odontogenic differentiation and bio-mineralization is an important characteristic of materials used in vital pulp therapy procedures.^5^ By having biocompatibility and sealing ability, MTA is the choice material in vivo, but addition of BO to the composite has resulted in some drawbacks.^9,11^ Therefore, recent studies have focused on introducing modified MTA or novel materials based on PC to circumvent these limitations by using various NPs and MPs on suitable scaffolds. In this regard, WPC has been accepted as an appropriate alternative material to improve biological and physicochemical characteristics of MTA.^22-24^ In the current experiment, the ratio of 20‒30 wt% of ZrO_2_ and ZnO MPs and NPs was used as a radiopacifier, with 70‒80% WPC, because of a higher radiopacity than that of dentin, approved by legal international committee ANSI/ADA.^18,21,25^

Based on MTT assay results, we found a positive effect of WPC on the viability of HDPSCs even after enrichment with ZrO_2_ and ZnO MPs and NPs over a period of 21 days. Consistent with this statement, it has previously been demonstrated that WPC enriched with ZrO_2_ NPs was successfully used in direct pulp cap procedures^15^ and better biological response was shown with these particles compared to BO in rat connective tissue.^26^ Notably, a negative effect of WPC containing ZnO NPs was revealed on the HDPSCs survival rate on day 7, while these effects reached near normal levels at the end stage of our study. One explanation would be that distinct concentration of ZnO and pH changes during cell culture at early stage could result in lower HDPSCs viability, but these effects decreased over time. Some studies have shown antioxidant and bactericidal activities of ZnO NPs while an excessive Zn concentration promoted ROS production and membrane dysfunction.^20,27^ Therefore, period, concentration and the route of administration must be carefully considered. Considering the positive effect of Zn NPs over time and the necessity of alkaline condition for the induction of dental mineralization, the use of ZnO particles and ZrO_2_ MPs/NPs could be applicable in the field of regenerative endodontics.^15,28^

ALP is a specific functional enzyme in the mineralization of dental tissue and induction of osteogenic/odontogenic differentiation of various SCs.^2^ We found that the combination of particles with WPC resulted in superior effects on the ALP activity, and NPs of ZnO with WPC were significantly better than MPs in this respect. Similar to the dynamics of ALP, the total calcium content also increased in all the groups as compared to the control cells pre-expanded on the plastic surface. Consistent with our data, it has been demonstrated that nanophase ZnO resulted in an enhanced ALP activity and calcium mineral deposition in comparison to microphase ZnO.^29^ It was previously reported that the treatment of mesenchymal SCs with ZnO NPs could induce osteogenic factors such as osteocalcin and ALP activity, and the production of extracellular matrix, mainly collagen.^30,31^ By providing an expanded surface area and incorporation of various membrane proteins in cultured cells via the application of ZnO NPs, it seems that the osteoblast differentiation would be accelerated by modulating ALP activity. Similar to the effect of ZnO particles, the films coated by zirconia exhibited a profound effect on osteoblast-like MG63 cells, ALP activity and the transcription level of osteogenic markers.^32^

According to a previous experiment, an increase in the level of ionized calcium could have promising effects on the orientation of intracellular osteogenic signaling pathway by engaging factor MAPK, while an alkaline pH is thought to promote the osteogenic behavior.^33^ The lower calcium content in WPC combined with nano/microparticles compared to WPC also could be related to the higher calcium content in pure WPC. Accordingly, the addition of a radiopacifying agent might restrict the release of calcium ions^21,34^ by reducing solubility but not by aborting cell functionality.^35^ However, in the current study, a combination of WPC with ZnO NPs resulted in increased calcium ion level contrary to others. By virtue of concomitant changes in the level of ALP and calcium, one could hypothesize that the mineralization and osteogenic capacity of cells increased on the surface of the scaffolds. With respect to these features, the higher calcium level in ZnO NPs might correlate with an increased solubility rate, possibly inducing the effect on target cells. Similar to ALP levels, calcium levels also diminished during 21-day incubation period. These results confirmed the close correlation between ALP and calcium.

## Conclusion

It can be concluded that WPC enriched with ZnO and ZrO_2_ particles induced osteogenic/odontogenic trans-differentiation by increasing ALP activity in vitro. Considering the results of the present study, further studies are needed for elucidating underlying interaction of HDPSCs with particles in vivo.

## Conflict of Interests

The authors declare no competing interests with regards to the authorship and/or publication of this article.

## Authors’ contributions

The concept and the design of the study were developed by SR, NGH and PD. The Isolation and culture of human dental pulp stem cells, were carried out by RR. The WPC disks were prepared and interpreted by BD. Data entry and statistical analyses, were carried out by SS and ASM. The manuscript was written by PD and RR. The proof reading was carried out by SR and SHSH. All the authors participated in the literature review.

## Acknowledgment

This study was a part of a PhD thesis and supported by a grant from Tabriz University of Medical Sciences. The authors would like to thank the Dental and Periodontal Research Center of Tabriz University of Medical Sciences and the personnel of the Stem Cell Research Center of Tabriz University of Medical Sciences.

## Funding

This research was carried out by financial support of the Dental and Periodontal Research Center, Tabriz University of Medical Sciences.

## Ethics approval

This research was approved by Research Ethics Commit-tee of Tabriz University of Medical Sciences in 2017.
